# Evaluation of Changes in Ocular Tissue Elasticity Before and After Trabeculectomy Using Shear Wave Elastography

**DOI:** 10.3390/jcm15093238

**Published:** 2026-04-24

**Authors:** Çiğdem Deniz Genç, Emre Aydın, Şaban Kılıç, Enes Gürün

**Affiliations:** 1 Department of Ophthalmology, Samsun University Faculty of Medicine, Samsun 55200, Türkiye; emreaydin0052@gmail.com; 2 Department of Ophthalmology, Samsun Training and Research Hospital, Samsun 55090, Türkiye; saban841kilic@gmail.com; 3 Department of Radiology, Samsun University Faculty of Medicine, Samsun 55200, Türkiye; e.grn06@gmail.com

**Keywords:** glaucoma, shear wave elastography (SWE), trabeculectomy

## Abstract

**Objective:** This study aimed to compare time-dependent changes in visual acuity (VA), central corneal thickness (CCT), intraocular pressure (IOP), and the structure and elasticity of ocular tissues, as measured by shear wave elastography (SWE), in the preoperative, postoperative 1st week, and 1st month periods. **Methods:** This prospective observational study with repeated measurements included 22 individuals aged 44–86 years who underwent trabeculectomy for medically resistant glaucoma. VA, CCT, and IOP were measured preoperatively and at 1 week and 1 month postoperatively for the study. The elastic properties of ocular tissues were evaluated using the SWE method by obtaining quantitative measurements of lens SWE (LSWE), vitreous SWE (VSWE), optic nerve head SWE (OSWE), and retrobulbar fat SWE (RSWE). The changes in the obtained structural and elasticity parameters were compared preoperatively, 1 week postoperatively, and 1 month postoperatively. **Results:** When anterior segment parameters were evaluated in individuals who underwent trabeculectomy, there were significant differences in VA (*p* = 0.001, ${ƞ}_{p}^{2}$ = 0.401), CCT (*p* = 0.001, ${ƞ}_{p}^{2}$ = 0.806), and IOP (*p* = 0.001, ${ƞ}_{p}^{2}$ = 0.853) values (*p* < 0.05). There was no statistically significant difference between preoperative and postoperative measurements at 1 week and 1 month for the elasticity parameters of LSWE, VSWE, OSWE, and RSWE (*p* > 0.05). **Conclusions:** This study suggests that the biomechanical adaptation of ocular tissues may be incomplete in the early postoperative period. Non-invasive methods such as SWE can be considered a potential tool for postoperative follow-up and predicting surgical success.

## 1. Introduction

Glaucoma is a group of progressive optic neuropathies characterized by degeneration of retinal ganglion cells and the retinal nerve fiber layer (RNFL), leading to changes at the optic nerve head, and is one of the leading causes of irreversible blindness worldwide [1]. In 2020, it accounted for 11% of all blindness cases worldwide among adults aged 50 and over [2]. In glaucoma treatment, a stepwise approach is widely adopted, prioritizing patient safety and starting with the least invasive options, progressing to more invasive and risky treatments when necessary [3]. These approaches are considered to be drug therapy and laser applications, respectively [4].

Trabeculectomy is a surgical procedure that creates a new drainage pathway in the eye to lower intraocular pressure (IOP). This reduction lowers the risk of optic nerve damage and slows glaucoma progression, making it the gold standard in surgical treatment [5,6]. Surgical success in trabeculectomy and other glaucoma surgeries is achieved by reducing postoperative IOP to a specific target range and to a sufficient degree [7]. However, fibrosis developing in the bleb area after trabeculectomy, increased tissue stiffness, and long-term structural changes can impair the function of the filtration bleb, negatively affecting the sustainability of surgical success [8,9]. Therefore, implementing fibrosis-preventing strategies and closely monitoring bleb morphology are critical for long-term surgical success [10,11]. Biomicroscopic examination and morphological classification systems are widely used after trabeculectomy [12]. However, these methods may vary among observers and may not directly reflect postoperative biomechanical properties [13]. Therefore, more reliable and objective measurement techniques are needed to predict surgical success [14].

Elastography is a non-invasive imaging method that evaluates the softness or hardness properties of tissues qualitatively or quantitatively and is used in addition to traditional gray-scale ultrasonography [15]. This method uses two main techniques: strain elastography (SE), which involves applying external pressure to tissues, and shear wave elastography, which is performed using waves generated by transducers (SWE) [16]. SWE, a real-time quantitative elasticity imaging technique, has recently been introduced, and successful studies have been reported in the breast, thyroid, liver, and musculoskeletal systems [17,18]. SWE is also used to evaluate the elastic properties of intraocular tissues and to examine the biomechanical characteristics of structures such as the lens [19], vitreous [20], and optic nerve head [21]. Compared to other imaging methods, it has distinctive advantages such as affordability, ease of use, lack of radiation, and real-time imaging [22]. The SWE method has been effectively used to evaluate the elasticity of pig and rabbit corneas, yielding successful results in animal models in the literature [17,23]. Also, studies of the cornea, sclera, and orbital tissues demonstrate that elastography is applicable for evaluating their biomechanical properties [24]. The use of elastography in ophthalmology is still limited. In particular, there are very few studies in the literature that evaluate ocular structures using SWE following trabeculectomy.

This study aims to reveal biomechanical changes in ocular structures by evaluating ocular structures before and after trabeculectomy using SWE. Findings obtained using this method may contribute to predicting surgical success. For this purpose, differences in IOP, corneal thickness, and SWE values of ocular structures during the preoperative and postoperative periods were investigated.

## 2. Materials and Methods

### 2.1. Participants

The study included the eyes of 22 open-angle glaucoma patients aged 44–86 who underwent trabeculectomy. The study was conducted prospectively at Samsun Training and Research Hospital between 1 June 2025 and 1 September 2025. The optimal number of subjects to participate in the study was determined using the GPower 3.1.3 program. The results indicated that a total of 16 subjects would be an appropriate sample size for the study (effect size r: 0.87, lower and upper critical *p*: 0.53, true power: 0.92). Although our enrolled sample of 22 patients exceeded the required sample size for the primary structural and clinical outcomes, it may have been underpowered to detect more subtle, delayed biomechanical variations in secondary elasticity parameters. Measurements were performed monocularly, focusing solely on the study eye scheduled for trabeculectomy. Participants were informed in detail before the study and included by providing a “Volunteer Consent Form.”

### 2.2. Study Design

This prospective observational study with repeated measurements was approved by the Samsun University Scientific Research and Publication Ethics Committee (approval number: 2025/7/15) and conducted in accordance with the Declaration of Helsinki. The inclusion criteria were a diagnosis of open-angle glaucoma, scheduled for trabeculectomy, and no history of glaucoma-related surgical intervention (tube shunt surgery, canaloplasty, or laser trabeculoplasty). Patients with a spherical equivalent greater than ±3.0 diopters were excluded from the study to ensure the homogeneity of the study cohort and to eliminate potential refractive confounders on ocular biomechanics. Additionally, those with an axial length of the globe >26 mm, patients with diseases such as uveitis, those with a history of ocular trauma, and patients with amblyopia in either eye were also excluded.

### 2.3. Procedures

All participants underwent comprehensive ophthalmological evaluations. Visual acuity (VA) (Snellen) and refraction assessments, slit lamp biomicroscopy, central corneal thickness (CCT) (Topcon CT-1P DRI-OCT-1 Triton, Tokyo, Japan) measurement, and detailed fundus examination after pupil dilation with 1% topical tropicamide (Tropamid Fort 1%, Bilim İlaç, Istanbul, Turkey) were performed. All evaluations were performed by a single experienced ophthalmologist to ensure measurement reliability and minimize observer-related variability. All measurements were repeated preoperatively and at 1 week and 1 month postoperatively, and were taken between 9:00 a.m. and 11:00 a.m. to avoid confounding effects of known diurnal fluctuations. IOP (Goldmann applanation tonometry; Haag-Streit, Koeniz, Switzerland) values were also recorded at these time points. At each measurement time point, eye elasticity measurements were obtained using B-mode ultrasound and sonoelastography.

### 2.4. B-Mode Ultrasonography and Sonoelastography Protocol

**Figure 1:**
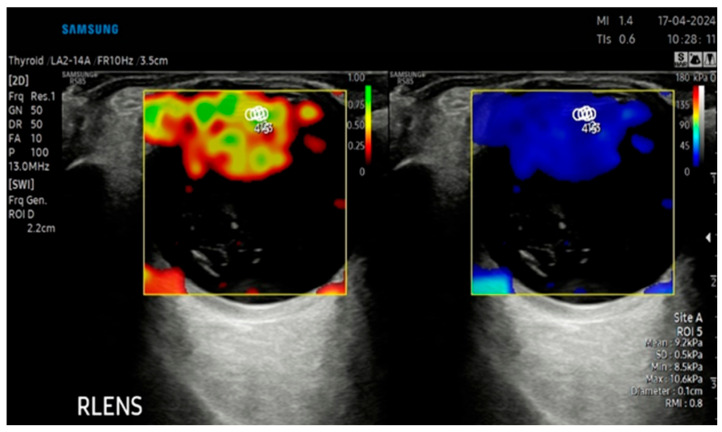
SWE measurements recorded in kilopascals (kPa), accompanied by an automatically calculated RMI (**left**) and the corresponding SWE maps (**right**) for the crystalline lens (CL).

**Figure 2:**
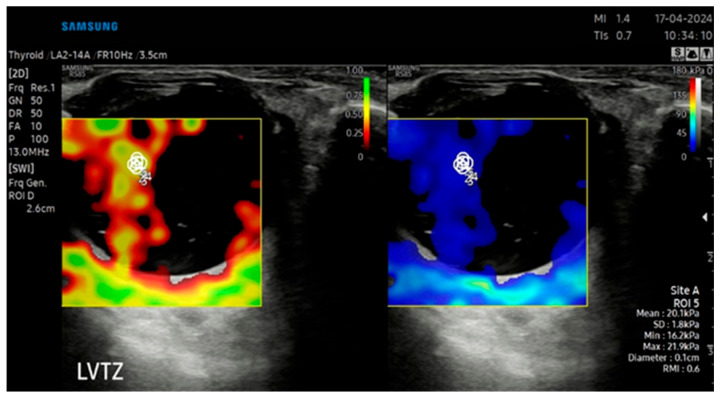
SWE measurements recorded in kPa, accompanied by an automatically calculated RMI (**left**) and the corresponding SWE maps (**right**) for the posterior vitreous (PV).

**Figure 3:**
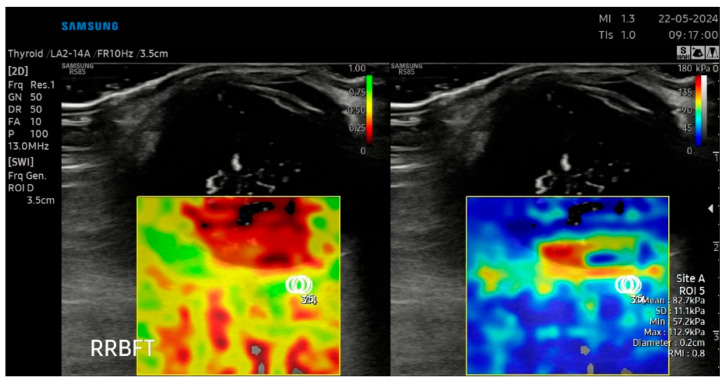
SWE measurements recorded in kPa, accompanied by an automatically calculated RMI (**left**) and the corresponding SWE maps (**right**) for the retrobulbar fat (RF).

**Figure 4:**
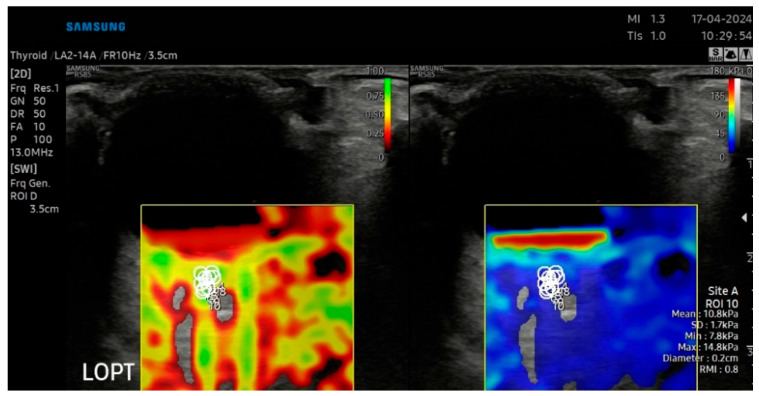
SWE measurements recorded in kPa, accompanied by an automatically calculated RMI (**left**) and the corresponding SWE maps (**right**) for the optic nerve (ON).

B-mode ultrasound and sonoelastography were performed for all patients using an RS-85 ultrasound device with a high-frequency linear transducer (2–14 MHz) (Samsung Medison Co., Ltd., Seoul, Republic of Korea) by the same radiologist with 8 years of US elastography experience. All measurements were taken with the patient lying on their back, and the eyelids were gently closed during the procedure. The patient’s head was maintained in a neutral midline position during all examinations, and the eye was kept still without voluntary ocular movement. To avoid direct probe–eyelid contact and to minimize undue pressure on the globe, a sufficient amount of ultrasound gel was applied over the closed upper eyelid. We evaluated ocular and periocular elasticity using SWE. The default scanner settings have been applied in the Brightness Mode (B Mode) window. A circular region of interest (ROI) of 1–2 mm was used during measurements. The ROI was positioned at the anatomical center of each target structure, namely the central part of the intraocular lens (IOL), the mid-posterior vitreous cavity (PV), the retrobulbar fat (RF) immediately posterior to the globe, and the anterior segment of the optic nerve head (ONH), with care taken to avoid adjacent tissues and anatomical borders Measurements were expressed simultaneously in meters per second (m/s) and kilopascals (kPa). The images were digitally stored in the ultrasound unit for later review. In all cases, the largest available sonoelastography box was used, including transverse scans of the IOL, PV, RF, and ONH. Sonoelastography images were displayed in real time by overlaying color-coded elasticity maps onto B-mode images using split-screen technology (Figure 1, Figure 2, Figure 3 and Figure 4). On the left side of the screen, the B-mode image and the Reliability Measurement Index (RMI) map are displayed, while the right side shows the elastogram.

To obtain consistent results, minimal pressure was applied during the examinations, and the average probe movement speed was maintained at approximately once per second. The wave line at the bottom of the screen was monitored to ensure adequate compression quality. The procedure was repeated until stable images were obtained, and image sequences of at least 15–20 s were recorded for each case. The color scale for qualitative evaluation ranged from red (high elasticity) to blue (low elasticity). The RMI, a device-specific performance index calculated as the weighted sum of the wave equation residual and the shear wave magnitude, was used as a quality-control parameter. According to the manufacturer, an RMI value ≥ 0.4 was considered acceptable, while 1.0 indicated excellent reliability and repeatability. For each structure at each time point (preoperative, postoperative 1st week, and postoperative 1st month), three SWE measurements with an RMI value of ≥0.4 were obtained from the same image, and the mean of these measurements was used for statistical analysis. Consequently, no recorded measurements were excluded from the study, as all documented data met the quality threshold.

### 2.5. Trabeculectomy Protocol

All surgical procedures were performed by the same surgeon under local anesthesia using the standard trabeculectomy technique. A standard fornix-based trabeculectomy was performed under local anesthesia. Briefly, a 5 × 4 mm scleral flap was created, and Mitomycin-C (Misintu 20 mg, Intas Pharmaceuticals, Ahmedabad, India) was applied subconjunctivally and under the scleral flap for 1 min using sponges, followed by extensive irrigation with at least 300 mL of balanced salt solution. A 3 × 1 mm trabecular block was excised, and a surgical peripheral iridectomy was performed. The scleral flap and conjunctiva were meticulously closed with appropriate sutures, and the procedure concluded with subconj bevacizumab (Avastin, Genentech, South San Francisco, CA, USA) and dexamethasone injections. A combination of moxifloxacin and dexamethasone was used for 1 week after surgery, followed by dexamethasone drops 4 times a day for 3 weeks. Topical cycloplegic agents were administered to patients three times daily for one week. Antiglaucomatous treatment was discontinued immediately after surgery. Treatment was adjusted based on postoperative IOP values. On the first postoperative day, in patients without bleb formation, attempts were made to create a bleb by applying pressure with the tip of a forceps on the scleral flap edge or by performing globe massage. Postoperatively, IOP, the appearance of the filtration bleb, anterior chamber depth, the presence of leakage assessed by the Seidel test, and any complications were recorded. Patients were monitored on the first day, first week, and first month. Maintaining IOP ≤ 21 mmHg postoperatively, with or without the addition of antiglaucomatous treatment, was considered a success; conversely, an IOP above 21 mmHg and/or the requirement for additional glaucoma surgery for a second surgical intervention was considered a failure.

### 2.6. Statistical Analysis

The SPSS program (SPSS for Windows, version 23.0, SPSS Inc., Chicago, IL, USA) was used for statistical analyses. The data are presented as mean and standard deviation. The Shapiro–Wilk test was applied for normality, the Levene test for homogeneity, and the data were found to be normally distributed. Repeated-Measures ANOVA was used to evaluate the preoperative, postoperative first-week, and postoperative first-month results of ocular structural parameters and elasticity parameters. Partial eta values were calculated to determine effect sizes. Statistical results were evaluated at a 95% confidence interval and a significance level of *p* < 0.05.

## 3. Results

Table 1 summarizes the participants’ descriptive characteristics.

**Figure 5:**
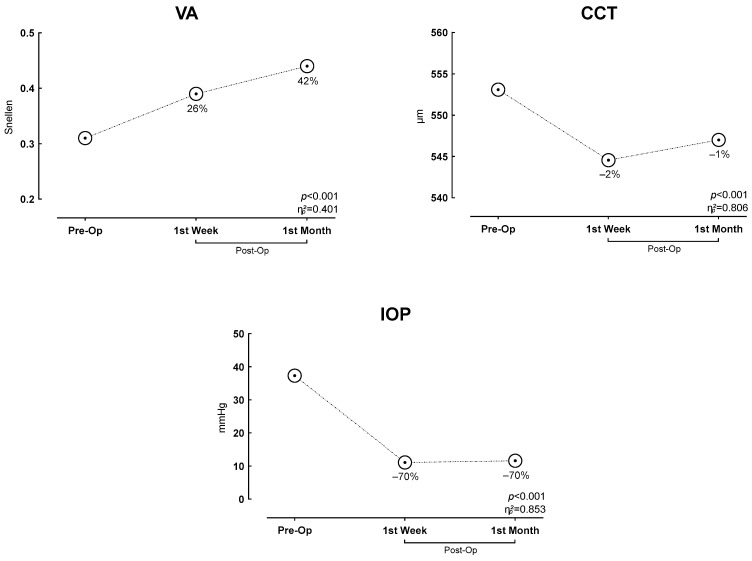
Changes in Time-Dependent Structural Parameters of the Eye.

Table 2 shows the comparison of eye segment structure values before and after the intervention. Post hoc pairwise comparisons revealed significant improvements across all structural parameters. The preoperative VA parameter significantly increased both at the first-week measurement (absolute increase of 0.08; *p* = 0.004, 95% CI: −0.137 to −0.029) and at the first-month measurement (absolute increase of 0.13; *p* < 0.001, 95% CI: −0.192 to −0.071). For CCT, a significant mean absolute reduction of 8.54 µm was observed at the first week (*p* < 0.001, 95% CI: 7.13 to 9.96), with a sustained reduction at the first month (*p* < 0.001, 95% CI: 4.64 to 7.54). Most notably, IOP demonstrated a dramatic 70.2% reduction from baseline at the first week (*p* < 0.001, 95% CI: 21.43 to 31.02) and maintained a 68.9% reduction at the first month (*p* < 0.001, 95% CI: 20.78 to 30.68) (Figure 5).

**Figure 6:**
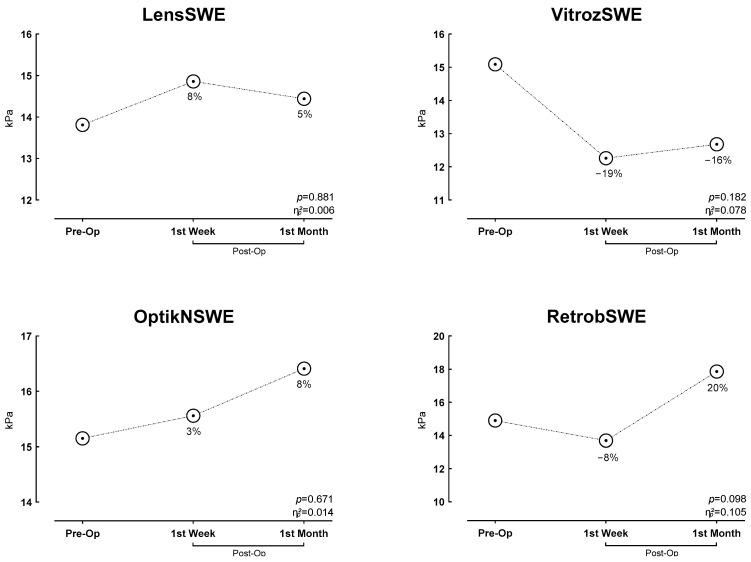
Change in Time-Dependent Elasticity Parameters.

Table 3 shows the comparison of elasticity values before and after the intervention. When the results were evaluated, the LSWE (*p* = 0.881, PES: 0.006), VSWE (*p* = 0.182, PES: 0.078), OSWE (*p* = 0.671, PES: 0.014), and RSWE (*p* = 0.098, PES: 0.105) parameters were not statistically significant (*p* > 0.05) (Figure 6). Although no statistically significant changes were observed in the elasticity parameters, certain observational trends were noted. Specifically, VSWE showed a slight decrease at the 1st week postoperatively, while RSWE demonstrated a mild upward trend by the 1st month. These variations may suggest subtle, time-dependent biomechanical shifts in the periocular environment following the surgical reduction of IOP.

## 4. Discussion

In our study, we evaluated the time-dependent changes in ocular structural and elasticity parameters in cases undergoing trabeculectomy. The major findings of the study were as follows: Significant differences were observed in VA, CCT, and IOP parameters at early (1 week) and short-term (1 month) follow-up compared with the preoperative period. When comparing elasticity parameters, the results before and after the intervention were similar for LSWE, VSWE, OSWE, and RSWE values. Current results suggest that the biomechanical adaptation of ocular tissues may not yet be complete in the early period following trabeculectomy.

The literature reports significant morphological and biomechanical changes in ocular structures after trabeculectomy [25]. Findings such as increased choroidal thickness and volume, shortening of axial length, and remodeling at the optic nerve head can be closely associated with postoperative IOP reduction [26]. These changes may be due to a decrease in intraocular pressure, leading to reduced ocular wall tension and stress on the lamina cribrosa [27]. Reference [28] stated that glaucoma patients who underwent trabeculectomy had lower IOP values and similar quality of life compared to those receiving medication. Reference [29] reported a significant decrease in IOP during the first month after trabeculectomy. Another study showed that, in cases of unilateral trabeculectomy, IOP decreased significantly in the operated eye during the first week after surgery [30]. The decrease in IOP after trabeculectomy can be explained by the diversion of aqueous humor to an alternative drainage pathway via the filtration bleb created by surgery and the reduction in scleral resistance [31]. The significant IOP reduction achieved in our study is consistent with the expected effect in the literature and demonstrates that surgery provides effective pressure control in the early period. It has been reported that a decrease in IOP reduces mechanical stress on the retina and optic pathway by decreasing the pressure load in the anterior chamber. Consequently, this mechanical relief contributes to improvements in VA and microcirculation in the early postoperative period [32]. Similarly, in our study, the significant increase in VA values in the early postoperative period may be associated with functional improvement in visual acuity due to decreased IOP [33]. The decrease observed in CCT after trabeculectomy can be explained by the resolution of postoperative edema, changes in the epithelial–endothelial balance, and temporary anterior segment changes [34].

Given tissue stiffness changes associated with IOP decline, elasticity parameters were evaluated using SWE in the early postoperative period in our study, and no statistically significant change in tissue elasticity was observed. This suggests that postoperative remodeling is still limited in the early stages [35,36]. It is necessary to evaluate why tissue elasticity did not show significant changes despite a major reduction in IOP [37]. While the immediate pressure drop relieves the mechanical load on the ocular walls, true alterations in tissue stiffness require a time-dependent process involving cellular remodeling, collagen fiber reorganization, and extracellular matrix adaptation [37,38]. Existing literature on ocular elastography suggests that outer, direct load-bearing structures like the cornea and sclera exhibit more rapid and measurable biomechanical responses to IOP fluctuations [38]. In contrast, deeper intraocular structures such as the lens and vitreous may experience delayed secondary biomechanical shifts. Furthermore, the lack of measurable elasticity changes may stem from specific technical limitations of SWE when applied to avascular tissues. The unique crystalline structure of the lens and the highly hydrated viscoelastic nature of the vitreous can differently influence shear wave propagation and attenuation [39,40]. Current SWE technology may lack the microscopic sensitivity required to detect extremely subtle, early biomechanical variations in these heterogeneous avascular media, meaning their intrinsic baseline stiffness could mask minor postoperative changes [41,42]. Recent literature further emphasizes that spatial resolution limits and motion artifacts in current elastography devices pose significant challenges for accurately evaluating microstructural intraocular tissues [43,44]. From a clinical perspective, SWE holds promise as a novel ‘biomechanical biomarker’ in routine postoperative care. Combining SWE with structural imaging, such as OCT, could help clinicians monitor whether IOP reduction translates into actual biomechanical stress relief in the optic nerve. Furthermore, tracking longitudinal elasticity in periorbital tissues may identify early subclinical fibrosis, allowing surgeons to tailor topical steroid regimens before clinical bleb failure occurs.

This study has some limitations. First, because the follow-up period was limited to the early postoperative period, biomechanical changes that may occur later could not be evaluated. Another limitation is the relatively small sample size and the single-center design of the study, which limit the generalizability of the findings. Although the current sample size provides sufficient statistical power to demonstrate significant improvements in primary clinical outcomes such as GIB, VA, and CCT, it poses a significant limitation for evaluating elasticity parameters. Since the study is likely underpowered to detect subtle biomechanical variations in the early postoperative period, the absence of statistically significant changes in tissue elasticity should be interpreted with caution. Therefore, the risk of a Type II error must definitely be considered for these specific negative findings. The study sample consists of 22 individuals aged 44–86. This limits the generalizability of the findings, as the sample size was small and the study was conducted at a single center. Technical factors, such as eye movements, probe angle, and tissue depth, that affect SWE measurements may have limited the accuracy of the results. Furthermore, the absence of a healthy control group and the monocular design of our study (evaluating only the operated eye without comparison to the contralateral unoperated eye) may have made it difficult to fully differentiate surgery-specific biomechanical changes from systemic physiological variations. Considering these limitations, studies with larger samples, multiple centers, and long-term follow-up can be conducted.

## 5. Conclusions

The results of the study showed a significant decrease in IOP after surgery, accompanied by changes in VA and CCT. The results were similar in the elasticity parameters evaluated with SWE. This indicates that the remodeling process in the eye tissues may not have started yet or may be limited in the early postoperative period. The results indicate that trabeculectomy provides effective pressure control in the early period, but changes in tissue elasticity may occur in later periods. Large-scale, multicenter prospective studies can describe the temporal and structural characteristics of biomechanical adaptation after trabeculectomy in greater detail. Also, the potential role of non-invasive imaging methods such as SWE in this process can make significant contributions to postoperative monitoring and personalized treatment approaches. In the literature, the number of studies evaluating ocular tissue elasticity using SWE after trabeculectomy is limited, and available data suggest that further research is needed to support findings in this field.

## Figures and Tables

**Table 1:** Descriptive of the groups (*n* = 22).

Variables	Mean	SD	Min	Max
Age (year)	64.23	11.14	44.0	86.0
Height (cm)	157.59	17.40	86.0	175.0
Weight (kg)	79.50	10.74	60.0	115.0
BMI (kg/m^2^)	28.49	3.86	24.22	37.98

SD, standard deviation; Min, minimum; Max, maximum; BMI, body mass index.

**Table 2:** Changes in Time-Dependent Structural Parameters of the Eye.

Variables	Pre-Op	1st Week	1st Month	*p*	${ƞ}_{p}^{2}$
	Mean ± SD	Mean ± SD	Mean ± SD		
VA (Snellen)	0.31 ± 0.238 ^a^	0.39 ± 0.240 ^b^	0.44 ± 0.273 ^b^	<0.001 *	0.401
CCT (µm)	553.09 ± 16.58 ^a^	544.55 ± 16.12 ^b^	547.00 ± 16.56 ^c^	<0.001 *	0.806
IOP (mmHg)	37.32 ± 10.50 ^a^	11.09 ± 1.27 ^b^	11.60 ± 1.53 ^b^	<0.001 *	0.853

* *p* < 0.05; SD, standard deviation; ${ƞ}_{p}^{2}$ partial eta square effect value; VA, visual acuity; CCT, central corneal thickness; IOP, intraocular pressure. Different superscript letters (a, b, c) in the same row indicate a statistically significant difference between the time points (*p* < 0.05).

**Table 3:** Change in Time-Dependent Elasticity Parameters.

Variables	Pre-Op	1st Week	1st Month	*p*	${ƞ}_{p}^{2}$
	Mean ± SD	Mean ± SD	Mean ± SD		
LSWE (kPa)	13.81 ± 6.54	14.86 ± 6.77	14.44 ± 7.04	0.881	0.006
VSWE (kPa)	15.09 ± 6.80	12.26 ± 6.11	12.68 ± 5.67	0.182	0.078
OSWE (kPa)	15.15 ± 8.80	15.56 ± 8.86	16.41 ± 7.45	0.671	0.014
RSWE (kPa)	14.90 ± 8.88	13.69 ± 7.18	17.86 ± 8.25	0.098	0.105

SD, standard deviation; ${ƞ}_{p}^{2}$ partial eta square effect value; LSWE, Lens Shear Wave Elastography; VSWE, Vitreous Shear Wave Elastography; OSWE, Optic Nerve Shear Wave Elastography; RSWE, Retrobulbar Shear Wave Elastography.

## Data Availability

The datasets generated and analyzed during this study are available from the corresponding author upon reasonable request.

## Abbreviations

VA	visual acuity
CCT	central corneal thickness
IOP	intraocular pressure
SWE	shear wave elastography
RNFL	retinal nerve fiber layer
ROI	region of interest
IOL	intraocular lens
PV	posterior vitreous
RF	retrobulbar fat
ONH	optic nerve head
m/s	meters per second
kPa	kilopascals
RMI	Reliability Measurement Index
SE	strain elastography

## References

[1] Allison K., Patel D., Alabi O. (2020). Epidemiology of glaucoma: The past, present, and predictions for the future. Cureus.

[2] Sun Y., Chen A., Zou M., Zhang Y., Jin L., Li Y., Zheng D., Jin G., Congdon N. (2022). Time trends, associations and prevalence of blindness and vision loss due to glaucoma: An analysis of observational data from the Global Burden of Disease Study 2017. BMJ Open.

[3] Micheletti J.M., Shultz M., Singh I.P., Samuelson T.W. (2025). An Emerging Multi-mechanism and Multi-modal Approach in Interventional Glaucoma Therapy. Ophthalmol. Ther..

[4] Funke C.M., Ristvedt D., Yadgarov A., Micheletti J.M. (2025). Interventional glaucoma consensus treatment protocol. Expert Rev. Ophthalmol..

[5] Besinis D. (2022). Trabeculectomy indications–when to do it (Trab vs MIGS). Acta Ophthalmol..

[6] Bell K., Bezerra B.D.P.S., Mofokeng M., Montesano G., Nongpiur M.E., Marti M.V., Lawlor M. (2021). Learning from the past: Mitomycin C use in trabeculectomy and its application in bleb-forming minimally invasive glaucoma surgery. Surv. Ophthalmol..

[7] Baker N.D., Barnebey H.S., Moster M.R., Stiles M.C., Vold S.D., Khatana A.K., Flowers B.E., Grover D.S., Strouthidis N.G., Panarelli J.F. (2021). Ab-externo MicroShunt versus trabeculectomy in primary open-angle glaucoma: One-year results from a 2-year randomized, multicenter study. Ophthalmology.

[8] Jiang K., Chen J., Tai L., Liu C., Chen X., Wei G., Lu W., Pan W. (2020). Inhibition of post-trabeculectomy fibrosis via topically instilled antisense oligonucleotide complexes co-loaded with fluorouracil. Acta Pharm. Sin. B.

[9] Sung M.S., Eom G.H., Kim S.J., Kim S.Y., Heo H., Park S.W. (2018). Trichostatin A ameliorates conjunctival fibrosis in a rat trabeculectomy model. Investig. Ophthalmol. Vis. Sci..

[10] Teus M.A., Paz Moreno-Arrones J., Castaño B., Castejon M.A., Bolivar G. (2019). Optical coherence tomography analysis of filtering blebs after long-term, functioning trabeculectomy and XEN^®^ stent implant. Graefe’s Arch. Clin. Exp. Ophthalmol..

[11] Sim J.J., Betzler B.K., Dorairaj S., Dada T., Ang B.C. (2025). Trabeculectomy Bleb Characteristics in Relation to Bleb Success Using Anterior Segment Optical Coherence Tomography–A Systematic Review and Meta-Analysis. J. Glaucoma.

[12] Singh M., Chew P.T., Friedman D.S., Nolan W.P., See J.L., Smith S.D., Zheng C., Foster P.J., Aung T. (2007). Imaging of trabeculectomy blebs using anterior segment optical coherence tomography. Ophthalmology.

[13] Tan J.C., Roney M., Posarelli M., Ansari A.S., Batterbury M., Vallabh N.A. (2025). Discriminatory power of trabeculectomy bleb internal reflectivity and morphology in surgical success using anterior segment optical coherence tomography. BMC Ophthalmol..

[14] Wlaz A., Kuna A., Wilkos-Kuc A., Rozegnał-Madej A., Aung T., Żarnowski T. (2020). Predictive value of bleb vascularity after mitomycin C augmented trabeculectomy. J. Clin. Med..

[15] Chamming’s F., Mesurolle B., Antonescu R., Aldis A., Kao E., Thériault M., Omeroglu A., Pinel-Giroux F., Seidler M., Solorzano S. (2019). Value of shear wave elastography for the differentiation of benign and malignant microcalcifications of the breast. Am. J. Roentgenol..

[16] Bamber J., Cosgrove D., Dietrich C.F., Fromageau J., Bojunga J., Calliada F., Cantisani V., Correas J.-M., D’Onofrio M., Drakonaki E.E. (2013). EFSUMB guidelines and recommendations on the clinical use of ultrasound elastography. Part 1: Basic principles and technology. Ultraschall Med.-Eur. J. Ultrasound.

[17] Tanter M., Bercoff J., Athanasiou A., Deffieux T., Gennisson J.-L., Montaldo G., Muller M., Tardivon A., Fink M. (2008). Quantitative assessment of breast lesion viscoelasticity: Initial clinical results using supersonic shear imaging. Ultrasound Med. Biol..

[18] Evans A., Whelehan P., Thomson K., McLean D., Brauer K., Purdie C., Jordan L., Baker L., Thompson A. (2010). Quantitative shear wave ultrasound elastography: Initial experience in solid breast masses. Breast Cancer Res..

[19] Aydin E., Gurun E., Cakir I.M., Ozturk M., Basaran M., Kilic S., Genc C.D., Ozkan I., Erdogan D. (2025). Impact of intravitreal aflibercept on ocular and periocular tissue elasticity: A shear wave elastography study in diabetic retinopathy. BMC Med. Imaging.

[20] Dikici A.S., Mihmanli I., Kilic F., Ozkok A., Kuyumcu G., Sultan P., Samanci C., Yilmaz M.H., Rafiee B., Tamcelik N. (2016). In vivo evaluation of the biomechanical properties of optic nerve and peripapillary structures by ultrasonic shear wave elastography in glaucoma. Iran. J. Radiol..

[21] Qiang B., Xu Q., Hu A., Fang J., Shen C., Zhang Y., Wang J. (2024). Feasibility of shear wave elastography for evaluating lens stiffness in patients with age-related cataracts: A quantitative analysis. Heliyon.

[22] Ferraioli G., Tinelli C., Dal Bello B., Zicchetti M., Filice G., Filice C., Liver Fibrosis Study Group (2012). Accuracy of real-time shear wave elastography for assessing liver fibrosis in chronic hepatitis C: A pilot study. Hepatology.

[23] Zhang X., Wang Q., Lyu Z., Gao X., Zhang P., Lin H., Guo Y., Wang T., Chen S., Chen X. (2018). Noninvasive assessment of age-related stiffness of crystalline lenses in a rabbit model using ultrasound elastography. Biomed. Eng. Online.

[24] Masud A.A., Liu J. (2024). Ultrasonic surface acoustic wave elastography: A review of basic theories, technical developments, and medical applications. Med. Phys..

[25] Gedde S.J., Feuer W.J., Lim K.S., Barton K., Goyal S., Ahmed I.I., Brandt J.D. (2020). Primary Tube Versus Trabeculectomy Study Group. Treatment outcomes in the primary tube versus trabeculectomy study after 3 years of follow-up. Ophthalmology.

[26] Nakakura S., Oogi S., Terao E., Nagata Y., Fujisawa Y., Dote S., Ueda K. (2024). Changes in ocular biometry following PreserFlo MicroShunt implantation and trabeculectomy: A prospective observational study. Cureus.

[27] Kim I., Gu W.M., Jeong A., Cha S.C. (2020). Long-term longitudinal changes in choroidal thickness with intraocular pressure reduction after glaucoma surgery. J. Korean Ophthalmol. Soc..

[28] King A.J., Hudson J., Fernie G., Kernohan A., Azuara-Blanco A., Burr J., Homer T., Shabaninejad H., Sparrow J.M., Garway-Heath D. (2021). Primary trabeculectomy for advanced glaucoma: Pragmatic multicentre randomised controlled trial (TAGS). BMJ.

[29] Savastano A., Maiola E., Carlà M.M., Gambini G., Boselli F., Giannuzzi F., Rizzo C., Rizzo S. (2025). Removable regulating sutures during trabeculectomy for a safer and more effective intraocular pressure control. Eur. J. Ophthalmol..

[30] Aghayeva F.A., Chronopoulos P., Schuster A.K., Pfeiffer N., Hoffmann E.M. (2021). Inter-eye relationship of intraocular pressure change after unilateral trabeculectomy, filtering canaloplasty, or PreserFlo™ microshunt implantation. Graefe’s Arch. Clin. Exp. Ophthalmol..

[31] Roddy G.W., Sit A.J. (2022). Surgical advancement of Tenon’s layer during trabeculectomy improves bleb morphology. J. Glaucoma.

[32] Silve Silveira V.D., Lindenmeyer R.L.M., Pakter H.M., Skaat A., Lavinsky D., Oliveira M.M., Picceti E., Lavinsky J., Mello P.A.d.A., Lavinsky F.M. (2023). Optic nerve head changes after intraocular pressure-lowering glaucoma surgeries using optical coherence tomography. J. Glaucoma.

[33] Wang T., Ling Q., Shen B., Jia X. (2025). The strong correlation between visual function improvement and retinal microcirculation enhancement in glaucoma. Front. Med..

[34] Lee N.S.Y., Ong R.M., Ong K. (2023). Changes in corneal endothelial cell density after trabeculectomy. Eur. J. Ophthalmol..

[35] Sun M.G., Son T., Crutison J., Guaiquil V., Lin S., Nammari L., Klatt D., Yao X., Rosenblatt M.I., Royston T.J. (2022). Optical coherence elastography for assessing the influence of intraocular pressure on elastic wave dispersion in the cornea. J. Mech. Behav. Biomed. Mater..

[36] Ramier A., Eltony A.M., Chen Y., Clouser F., Birkenfeld J.S., Watts A., Yun S.H. (2020). In vivo measurement of shear modulus of the human cornea using optical coherence elastography. Sci. Rep..

[37] Czerpak C.A., Kashaf M.S., Zimmerman B.K., Quigley H.A., Nguyen T.D. (2023). The strain response to intraocular pressure decrease in the lamina cribrosa of patients with glaucoma. Ophthalmol. Glaucoma.

[38] Vranka J.A., Kelley M.J., Acott T.S., Keller K.E. (2015). Extracellular matrix in the trabecular meshwork: Intraocular pressure regulation and dysregulation in glaucoma. Exp. Eye Res..

[39] Ganeau A., Lafond M., Legrand F., Laloy-Borgna G., Ben Moussa O., Poinard S., Mascarelli F., Thuret G., Gain P., Lafon C. (2023). Characterization of the viscoelastic properties of in vitro crystalline lens samples using ultrasound elastography. Appl. Phys. Lett..

[40] Mekonnen T., Zevallos-Delgado C., Singh M., Aglyamov S.R., Larin K.V. (2023). Multifocal acoustic radiation force-based reverberant optical coherence elastography for evaluation of ocular globe biomechanical properties. J. Biomed. Opt..

[41] Hollerieth K., Gaßmann B., Wagenpfeil S., Moog P., Vo-Cong M.T., Heemann U., Stock K.F. (2016). Preclinical evaluation of acoustic radiation force impulse measurements in regions of heterogeneous elasticity. Ultrasonography.

[42] Lan G., Gu B., Larin K.V., Twa M.D. (2020). Clinical corneal optical coherence elastography measurement precision: Effect of heartbeat and respiration. Transl. Vis. Sci. Technol..

[43] Wang L., Zhu Y., Huang H., Huang L., Zhang Z., Luo N., Álvarez-Arenas T.E.G., Shi J., He X. (2026). Advances in the Application of Optical Coherence Elastography in Ophthalmology. J. Innov. Opt. Health Sci..

[44] Lan G., Twa M.D., Song C., Feng J., Huang Y., Xu J., Qin J., An L., Wei X. (2023). In vivo corneal elastography: A topical review of challenges and opportunities. Comput. Struct. Biotechnol. J..

